# Chaotic microlasers caused by internal mode interaction for random number generation

**DOI:** 10.1038/s41377-022-00890-w

**Published:** 2022-06-20

**Authors:** Chun-Guang Ma, Jin-Long Xiao, Zhi-Xiong Xiao, Yue-De Yang, Yong-Zhen Huang

**Affiliations:** 1 State Key Laboratory of Integrated Optoelectronics, Institute of Semiconductors, Chinese Academy of Sciences, Beijing, 100083 China; 2 Center of Material Science and Optoelectronic Engineering, University of Chinese Academy of Sciences, Beijing, 100049 China

**Keywords:** Nonlinear optics, Semiconductor lasers

## Abstract

Chaotic semiconductor lasers have been widely investigated for generating unpredictable random numbers, especially for lasers with external optical feedback. Nevertheless, chaotic lasers under external feedback are hindered by external feedback loop time, which causes correlation peaks for chaotic output. Here, we demonstrate the first self-chaotic microlaser based on internal mode interaction for a dual-mode microcavity laser, and realize random number generation using the self-chaotic laser output. By adjusting mode frequency interval close to the intrinsic relaxation oscillation frequency, nonlinear dynamics including self-chaos and period-oscillations are predicted and realized numerically and experimentally due to internal mode interaction. The internal mode interaction and corresponding carrier spatial oscillations pave the way of mode engineering for nonlinear dynamics in a solitary laser. Our findings provide a novel and easy method to create controllable and robust optical chaos for high-speed random number generation.

## Abstract (web summary)

Chaotic output of a self-chaotic microlaser with the typical wide lasing spectra, as shown, was applied for high speed random number generation.

## Graphical abstract

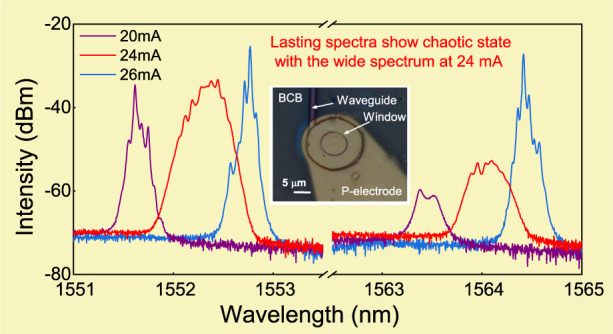

## Introduction

Random numbers are crucial in the generation of cryptographic keys for classical and quantum cryptography systems, the reliability of modern networked society, and stochastic simulation, etc.^1–4^. Pseudorandom numbers can be generated by using deterministic algorithm programs but with limited unpredictability, which reduces the randomness and associated security. Physical entropy sources, such as electronic and photon noise, thermal noise in resistors, and frequency jitter in oscillators, have been applied to generate non-deterministic truly random number sequences^5–7^. However, due to the low signal level associated with physical noise, non-deterministic random number sequence generation rates from stochastic processes are usually less than 100 Mbit s^−1^^8^. High-bandwidth chaotic semiconductor lasers have been widely investigated for the generation of random numbers^8–21^ and secure communications^22–24^. Based on decay rates of electric field, population inversion, and polarization, lasers were classified as class A, B, and C governed by one, two, and three equations, respectively^25^. The chaotic laser was first demonstrated without an external perturbation for a class C laser. As class B lasers, semiconductor lasers governed by two equations are hard to exhibit chaotic output without external perturbations. But semiconductor lasers are extremely sensitive to external perturbations because their lasing frequency is affected by carrier density. Chaotic semiconductor lasers were realized by using external optical feedback^8–14^ and optical injection^20,26^. Furthermore, chaotic behaviors were investigated for integrated semiconductor lasers with a passive feedback cavity, or with a mutual coupling integrated microlasers^15–19,27^.

Under continuous perturbations of an external optical injection or delayed optical feedback, semiconductor lasers can exhibit strong nonlinear dynamics, such as periodic oscillations and chaos. However, time-delay periodicity imprinted in the laser output leads to recurrences in the outcome from chaotic lasers with external optical feedback^28,29^, which reduces the randomness and security in random number generation, and post-processing is usually required to obtain truly random numbers. Moreover, the dynamical behaviors are sensitive to the parameters of the perturbations and precise adjustment is essential to realize specific nonlinear dynamics of interest. A chaotic solitary laser without external perturbations is a prominent configuration for random number generation due to its robust and simple scheme. Low-dimensional chaos in polarization-resolved output power was reported for a free-running quantum-dot vertical-cavity surface-emitting laser, which was generated by nonlinear mode competition including carrier spin relaxation^30,31^. Lasing of multiple transverse modes was demonstrated in wave-chaotic microcavity lasers for suppressing lasing instabilities^32^, and spatiotemporal interference of many lasing modes with stochastic spontaneous noises were used for massively parallel ultrafast random bit generations in a specially designed chip-scale laser^33^. In the past decades, deformed microcavities have attracted a great attention, especially for realizing direction emission microlasers, because of the transition of incident angles for mode light rays inside the microcavity^34^. Recently, the internal field patterns of different optical modes in deformed silicon microdisks were experimentally mapped for different dynamic states^35^. A phase-space tailoring scheme was reported to regulate photon transport in a chaotic microcavity, and control of far-field pattern was verified experimentally for a quantum-dot microlaser^36^. However, a chaotic mode is not directly related to chaotic output for a deformed microcavity laser.

In this article, we propose a novel approach to manipulate the temporal dynamics of a solitary semiconductor microlaser by nonlinearly coupling of two transverse modes inside the microcavity. Chaotic output is realized from the total output of the deformed microcavity laser without external optical or electric perturbations, which allows to form a simple, small, and robust random signal source. As in a microdisk resonator, polygonal resonators can also support high-Q whispering-gallery modes with the mode light rays undergoing total internal reflection. A dual-mode lasing square microlaser was demonstrated with an adjusted mode frequency interval^37^, and a circular-sided hexagonal resonator was designed to enhance mode *Q* factor^38^. Here, we design a circular-sided hexagonal microresonator (CSHM) to enhance passive mode *Q* factors and adjust the mode frequency interval. For a microcavity with high passive mode *Q* factors, we can realize dual-mode lasing for the fundamental and first order transverse modes with near threshold gain, mainly determined by the other losses, e.g., material absorption loss. In a dual-mode lasing microlaser, mode beating can lead to oscillations of the photon density and carrier density caused by stimulated emission, especially as the mode interval is close to the laser relaxation oscillation frequency. Moreover, the oscillation of the carrier density will result in side peaks for lasing modes as under external electric modulation, and lead to nonlinear coupling for the two lasing modes because the oscillation frequency is the frequency interval of the two lasing modes. Using a rate equation model including internal mode interaction, we predict nonlinear dynamic states including chaotic state for a dual-mode lasing microlaser. Furthermore, we fabricate CSHM lasers and demonstrate nonlinear dynamical states including period-oscillation states and chaotic state, using a solitary CSHM laser. The chaotic state is verified with a positive *K*_2_-entropy of *K*_2_ ≈ 2.9 ns^−1^, and physical random numbers at 10 Gb s^−^^1^ verified by statistical tests are obtained directly from the total output intensity of the microlaser. The solitary microcavity laser with chaotic total output intensity and period-oscillations provides a simple scheme for high-speed random number generation and the investigation of nonlinear dynamics.

## Results

### Chaotic principle for a dual-mode microlaser

**Fig. 1:**
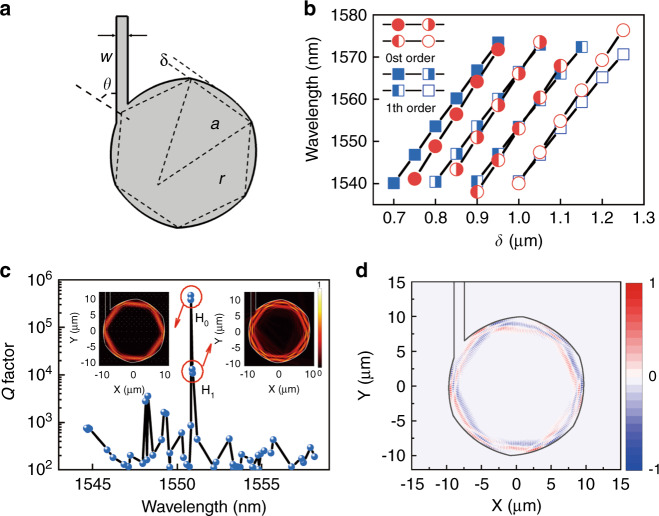
Simulated diagram and mode characteristics. **a** Schematic diagram of a CSHM with flat sides replaced by arc sides. **b** Mode wavelengths versus *δ* for *H*_0_ and *H*_1_ modes, indicated by circle and square symbols, respectively. The modes with the same longitudinal-mode numbers are indicted by the same symbols and connected by lines, for the CSHM with *a* = 10 μm and *w* = 1.5 μm. **c** Mode *Q* factors versus mode wavelengths at *δ* = 1.01 μm. Inset: Magnetic field (|*H*_*z*_|) distributions of mode *H*_0_ and mode *H*_1_. **d** Real part of cross coefficient distribution for the *H*_0_ and *H*_1_ mode fields, with positive and negative values in red and blue colors marked in-phase and antiphase sections

To realize dual-mode lasing with a tunable mode frequency interval, we choose a circular-side hexagon microcavity with a ring electrode. The circular-side can enhance mode *Q* factors for realizing dual mode lasing of the fundamental and first order transverse modes, and the deformation magnitude of circular-side can be used for adjusting mode interval to enhance nonlinear dynamics inside the microcavity. Furthermore, a ring-pattern electrode is designed for further adjusting the mode interval as for the square microcavity laser in^37^. A CSHM is simulated for transverse electric (TE) modes, i.e., the domination polarization for a compressively stressed laser wafer. As shown in Fig. 1a, a deformation amplitude *δ* is defined as $\delta = r - \sqrt {r^2 - \left( {a{{{\mathrm{/}}}}2} \right)^2}$, where *a* and *r* are the hexagonal flat-side length, the radius of the circular arc, and *w* and *θ* are the width of the output waveguide and the acute angle between the waveguide and the diagonal of the hexagon, respectively. The wavelengths of the fundamental and first-order transverse modes (*H*_0_ and *H*_1_) versus deformation amplitude *δ* are plotted in Fig. 1b, for the CSHM with *a* = 10 μm and *w* = 1.5 μm. The wavelengths of *H*_0_ and *H*_1_ redshift with *δ* at different rates and cross at about *δ* = 1.01 μm. In addition, a refraction index offset Δ*n* inside a ring with external and inside radii of 9.5 and 4.5 μm is applied to model the influence of a current injection window. As Δ*n* increases with injection current due to the temperature rising, the wavelength of *H*_0_ approaches to that of *H*_1_ and becomes longer eventually, which indicates the mode frequency interval can also be adjusted by injection current. The passive mode *Q* factors are plotted in Fig. 1c, where the mode *Q* factors of *H*_0_ and *H*_1_ are greater than 1 × 10^4^ suitable for dual-mode lasing, as the practical mode *Q* factors limited by material absorption loss are in the order of 10^3^. In addition, two degenerate modes with near mode *Q* factors are obtained for *H*_0_ and *H*_1_ respectively. The insets in Fig. 1c are *z*-direction magnetic field distributions |*H*_*z*0_| and |*H*_*z*1_| for *H*_0_ and *H*_1_ modes at the wavelengths of 1550.83 and 1550.92 nm, respectively. Based on the mode field distributions, we can define a cross coefficient proportional to *H*_*z*0_H*_z1_ as shown in Eq. (S2) (see the Supplementary Information). The real part of the cross coefficient is plotted in Fig. 1d, which indicates in-phase and antiphase sections of the mode fields in red and blue colors. The in-phase and antiphase sections will result in positive and negative mode beating intensities, and carrier density will oscillate at beating frequency of the two modes caused by the variation of stimulated emission. In the following, a rate equation model was setup for a dual-mode lasing microcavity laser with internal interaction including the oscillation of carrier density in the two sections due to the mode beating.

**Fig. 2:**
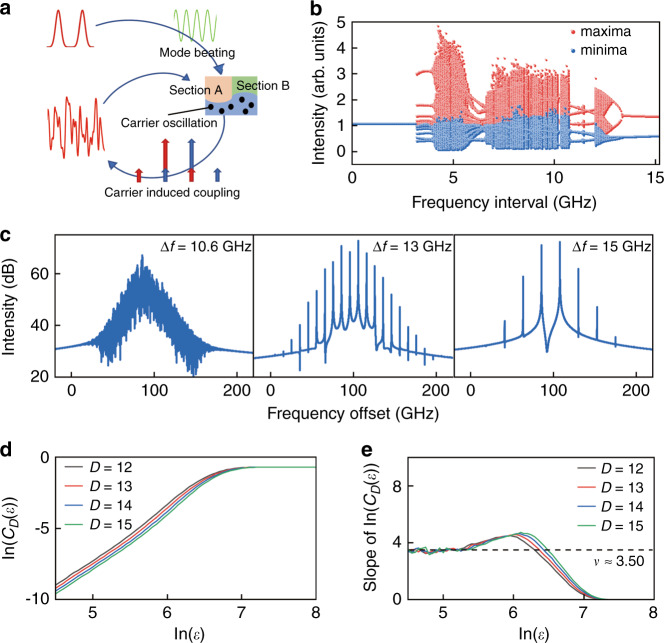
Schematic diagram and simulated results of the rate equation. **a** Schematical diagram of mode interaction, including mode beating intensity oscillation in the two sections, carrier oscillation caused by stimulated emission of beating intensity, the appearances of side peaks for lasing modes which can work as optical injection term for the other mode, and output power due to mode interaction. The long and small arrows represent the lasing modes and the side peaks caused by carrier oscillation. **b** Bifurcation of the extrema for the temporal output waveform versus dual-mode frequency interval. **c** Optical spectra of chaos, period-two, and period-one states at Δ*f* = 10.6, 13, and 15 GHz, respectively. **d** Simulated correlation integral curve of the *C*_*D*_(*ε*) versus the sphere radius *ε* with embedding dimension *D* = 12 to 15 at Δ*f* = 10.6 GHz, and **e** the slope of the integral curve is convergent to a correlation dimension *ν* ≈ 3.50

Different rate equation models were used to analyze nonlinear dynamics of a solitary laser system. For example, bistable operation and chaotic state were studied for a two-section semiconductor laser under sinusoidal current modulation, using a rate equation with inhomogeneous excitation and spatial distribution of carrier lifetime^39^. The complex nonlinear behaviors were caused by different parameters, such as lifetimes, of the two sections. In addition, complex periodic and chaotic intensity fluctuations were characterized for a solid-state laser with intra-cavity frequency doubling crystal using an intensity rate equation model^40^. Nonlinear dynamic behaviors of low-frequency mode intensity fluctuations were investigated using the rate equations with multiple modes in two orthogonal polarizations. But longitudinal mode beating phenomena were not accounted in the intensity equations. By using field equations, we set up a rate equation model including the influence of mode beating intensity and internal mode injections for a dual-mode lasing microlaser. As shown in Fig. 1d, the in-phase and anti-phase for the mode fields occupy about a half region, which will interchange at the mode frequency interval between *H*_0_ and *H*_1_. By simply dividing the microcavity into two sections of *A* and *B* with the same sign of the products, we expect the carrier densities in each section will oscillate with the beating optical intensity due to the consumption of stimulated emission. As shown in Fig. 2a, the mode beating intensity and carrier oscillation at the beating frequency will cause side peaks for lasing modes at the mode frequency interval and result in strong mode interactions. Accounting the internal mode interaction between *H*_0_ and *H*_1_ and dividing the microcavity into the *A* and *B* sections, we set up a comprehensive rate equation model for the CSHM laser similar as in^41,42^

1 $$\begin{array}{ll}\frac{{{\rm{d}}N^p}}{{{\rm{d}}t}}\!\!\!\! &= \frac{{\eta I}}{{qV}} - \frac{{N^p}}{{\tau _e}} - v_g\mathop {\sum}\limits_{m,m{^\prime} } {K_{mm{^\prime} }^pg^p{\mathrm{Re}} \left( {E_mE_{m{^\prime} }^ \ast } \right)}\qquad \left( {m,m{^\prime} = 0,1;p = A,B} \right)\\ \frac{{{\rm{d}}E_m\left( t \right)}}{{{\rm{d}}t}} \!\!\!&= - \frac{1}{{2\tau _m}}E_m\left( t \right) + \frac{1}{2}v_g\left( {1 + {\rm{i}}\alpha } \right)\mathop {\sum}\limits_{m{^\prime} } {\left( {K_{mm{^\prime} }^A\Gamma g^A + K_{mm{^\prime} }^B\Gamma g^B} \right)E_{m{^\prime} }\left( t \right)} \end{array}$$

where the slowly varying complex field of transverse mode *m* is $E_m\left( t \right) = \left| {E_m\left( t \right)} \right|\exp \left( {{\rm{j}}\Phi _m\left( t \right)} \right)$, the mode phase is $\Phi _m\left( t \right) = \phi _m\left( t \right) + \left( {\omega _m - \Omega } \right)t$ with a reference frequency Ω, and the mode frequency interval is $\Delta f = \left( {\omega _1 - \omega _0} \right)/2\pi$. *m* and *m*′ = 0 and 1 is corresponding to the fundamental and first order transverse mode, respectively, *τ*_*m*_ is mode lifetime determined by passive mode *Q* factor and internal absorption loss, *α* is the linewidth enhancement factor, *p* = *A* and *B* represents the two sections, *N*^*p*^, *g*^*p*^ and $K_{mm\prime }^p$ are carrier density, gain and the mode field overlap coefficient of mode *m* and *m*′ in section *p* (see Supplementary Information for more details).

The rate equations with the parameters (see Equation S1 and Table S1) were solved using a fourth order Runge–Kutta integration with a time step of 1 ps to generate the time series of the output photon density *S*, and Fourier transforms were used to produce the optical and radio frequency (RF) signal spectra. The extremum-bifurcation diagram in Fig. 2b, calculated by searching for the local maxima and minima of *S*, shows the internal mode injection locking with a constant intensity, as the mode frequency interval Δ*f* is smaller than 3 GHz, and strong mode interactions result in a great number of extrema as Δ*f* increases from 3 to 13 GHz. In addition to the chaotic region around resonance frequency *f*_*r*_ about 10 GHz, chaotic states appear around the frequency interval Δ*f* = 5 GHz. As shown in Fig. S1, the RF spectra have strong resonance peak at *f*_*r*_ as Δ*f* = 5 GHz ≈ *f*_*r*_ /2. The lasing spectra are shown in Fig. 2c at Δ*f* = 10.6, 13, and 15 GHz, corresponding to chaotic state, period-two, and period-one oscillations, respectively. The nonlinear dynamics change from complex to simple with the further increase of Δ*f* because the carrier density oscillates weakly as Δ*f* is much larger than the relaxation oscillation frequency.

**Fig. 3:**
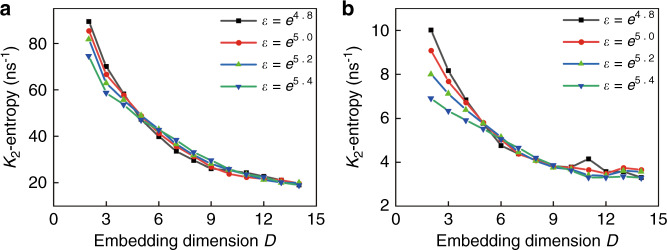
The entropy (*K*_2_) of the simulated time-series before and after a filtering process with 2 GHz bandwidth. **a** *K*_2_ = 19 ns^−1^ and **b** *K*_2_ = 3.2 ns^−^^1^ are obtained directly from the chaotic output series of the rate equations and accounting 2 GHz bandwidth of the ADC, respectively, at Δ*f* = 10.6 GHz

The simulated output intensities were evaluated using the Grassberger–Procaccia (G–P) algorithm to estimate the correlation dimension *ν* and the *K*_2_-entropy (Kolmogorov entropy)^43,44^. The output data can be categorized as periodic or quasiperiodic, chaotic, and purely stochastic signals when *K*_2_ is zero, positive, and infinity, respectively^43^. For the time series at Δ*f* = 10.6 GHz, the correlation integral *C*_*D*_(*ε*) shows a linear correlation with the radius of the ball *ε* at small values in logarithmic scale in Fig. 2d, and the corresponding slope of the curve converges to the correlation dimension of *ν* ≈ 3.50, as shown in Fig. 2e from *D* = 12 to 15. Taking into accounting 2 GHz bandwidth of ADC (analog-to-digital converter) as in the experiment, we get the correlation dimension *ν* of 3.91 in Fig. S2d. Furthermore, the *K*_2_-entropy of *K*_2_ = 19 and 3.2 ns^−^^1^ is derived directly using the chaotic output series of the rate equations at Δ*f* = 10.6 GHz and accounting 2 GHz bandwidth of ADC, respectively, as shown in Fig. 3, which verifies the chaotic characteristic of the time series.

### Experimental realization of a solitary chaotic microlaser

**Fig. 4:**
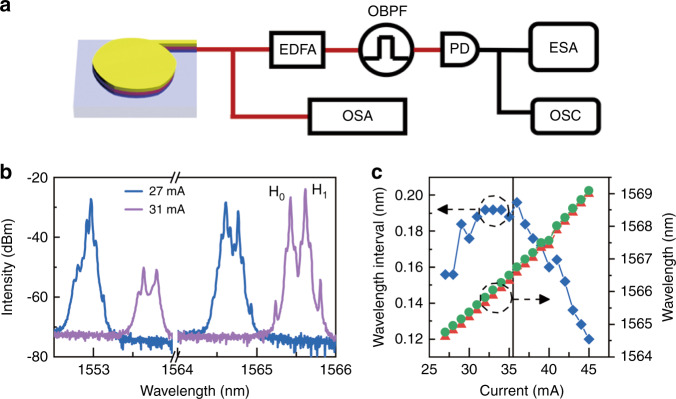
Experimental set up and lasing characteristics of the CSHM laser. **a** Schematic of the experimental setup for the nonlinear dynamic measurement and lasing spectra of the CSHM laser, where the optical output emitted from the CSHM is collected by a tapered single-mode fiber (SMF) and first measured by an optical spectrum analyzer (OSA) to get an overall estimation for the nonlinear dynamics. Alternatively, the SMF is connected to an EDFA to amplify the optical power. The optical signal is filtered by an optical band pass filter (OBPF) and then transformed to electrical signal by a photodetector (PD). The electrical spectrum and the temporal waveform are then measured by an electric spectrum analyzer (ESA) and an oscilloscope (OSC). **b** Detailing lasing spectra at *I* = 27 and 31 mA exhibiting the lasing mode hopping. **c** Lasing mode wavelengths and corresponding wavelength interval versus the injection current

**Fig. 5:**
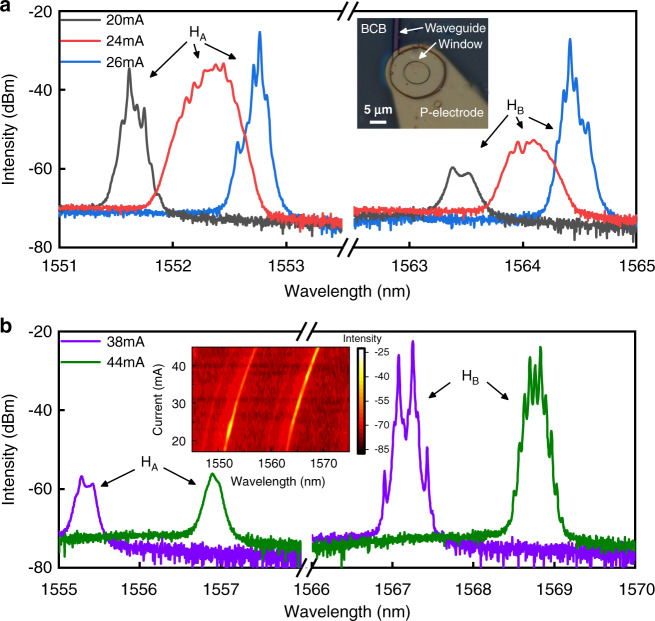
Characteristic lasing spectra of the CSHM laser. **a** Lasing spectra at 20, 24, and 26 mA to show the spectral broadening and mode hopping from a group *H*_A_ to *H*_B_; inset: the microscopic image of a CSHM laser. **b** Lasing spectra with main lasing modes in *H*_B_. Period-one and Period-two states can be observed clearly at 38 and 44 mA, respectively; inset: lasing spectra versus current, including two groups of modes *H*_A_ and *H*_B_ in the whole scale

A CSHM laser with *a* = 10 μm, *w* = 1.5 μm, *δ* = 1.01 μm and *θ* = 55° as shown in inset of Fig. 5a is tested at temperature 287 K controlled by a thermoelectric cooler, which has a ring-patterned contact window for selective electric injection to adjust the mode frequency interval^37^. Figure 4a shows the schematic of the experimental setup.

The lasing spectra at 27 and 31 mA are shown in Fig. 4b with the main lasing modes around 1553 and 1565.5 nm, respectively. The mode wavelengths of *H*_0_ and *H*_1_ and corresponding wavelength intervals are presented in Fig. 4c versus the injection current. The mode wavelengths increase with the injection current mainly due to the heating effect. But the wavelength interval first increases with the injection current from 24 to 29 mA caused by the increase of carrier density accompanying lasing mode jumping from about 1553 to 1565.5 nm. Then the wavelength interval keeps near constant from 30 to 36 mA due to the compensation of the increases of carrier density and temperature, and finally decreases with the injection current as it is larger than 36 mA mainly caused by heating effect. The wavelength interval decreases with the increasing current, indicating a larger redshift rate of the mode at shorter wavelength side. Taking the simulation results into account, we estimate that the mode at shorter wavelength side is the *H*_0_ mode, i.e., the mode wavelength interval in Fig. 4c is (λ_1_ − λ_0_).

Detailed lasing spectra are plotted in Fig. 5, which indicate complicated nonlinear dynamical states due to mode interaction and mode redshift. Near single-mode operation at short wavelength side is obtained at 20 mA with a side-mode suppression ratio about 10 dB. Four main peaks around 1552.3 nm with intensity differences less than 2 dB and mode frequency intervals of 4–9 GHz are observed at 24 mA in Fig. 5a, which can be assigned as the fundamental and first-order transverse modes including degenerate modes, and similar spectra are observed from 22 to 24 mA. More lasing mode will result in strong mode competition. In fact, it was found that dual-mode lasing state is easy to realize chaotic state than single mode lasing state under external optical feedback^45^. Multi-mode lasing with such small intervals can result in complicated nonlinear processes as predicted in Fig. 2. Two longitudinal modes marked as *H*_A_ and *H*_B_ with a free spectral range of about 12 nm have near the same intensity as shown in Fig. 5a and the inset of Fig. 5b at 26 mA, due to the redshift of the gain spectrum with the increase of injection current. At 38 mA, two main lasing modes around 1567.2 nm with a frequency interval 18.2 GHz are observed with two clear four-wave mixing (FWM) peaks in Fig. 5b, and additional peaks of period-two oscillation appear at 44 mA due to the small mode frequency interval of 16.5 GHz, as carriers can oscillate with a large amplitude at 16.5 GHz.

### Identification of chaos output

**Fig. 6:**
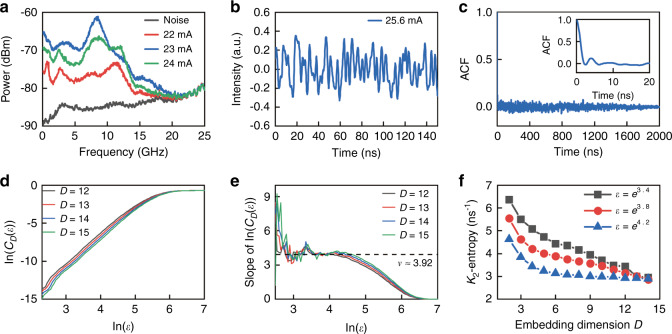
Chaotic output and statistics analysis for the CSHM laser. **a** Output RF spectra at 22, 23, and 24 mA and noise spectrum of the test system. **b** Temporal output waveform and **c** corresponding autocorrelation function at 25.6 mA, inset: zoom-in view of ACF. **d** The correlation integral curve of *C*_*D*_(*ε*) versus the radius of the ball *ε* in logarithmic scale, and **e** corresponding slope of the integral curve in (**d**). **f** Derived *K*_2_-entropy from the integral curve. All the curves tend to converge to a common value about *K*_2_ ≈ 2.9 ns^−1^ as *D*→∞

Furthermore, the output of the CSHM laser was amplified by an erbium-doped fiber amplifier (EDFA) and converted to an electrical signal by a 40 GHz-bandwidth photodetector, and the AC component with AC coupling was measured by an oscilloscope with 2 GHz-bandwidth and 8-bit ADC. The output radio-frequency (RF) spectra at *I* = 22, 23, and 24 mA are plotted in Fig. 6a with the noise spectrum measured at *I* = 0. The output RF spectra are noise like flat spectra with flatness within ±5 dB in the bandwidth of 11.6 GHz^46^. The output waveforms are measured using an oscilloscope at a sampling rate of 10 GSa s^−1^ and plotted in Fig. 6b at *I* = 25.6 mA, which shows noise-like intensity oscillations with an autocorrelation function (ACF) as shown in Fig. 6c. The ACF has the full width at half maximum of 1 ns and exhibits no specific sidelobes in a long range of 2 μs, which are different from optical feedback chaotic lasers^45^.

**Fig. 7:**
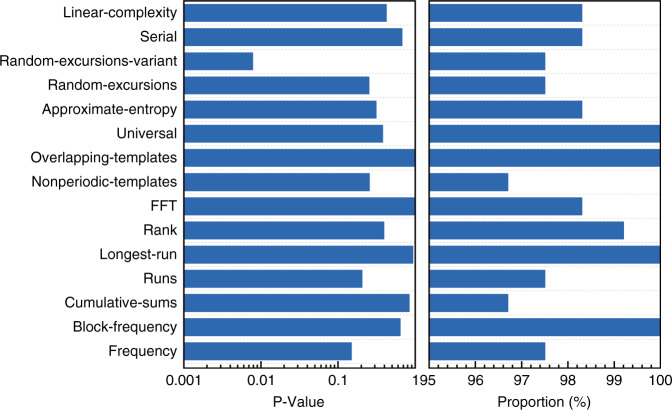
Results of the NIST Special Publication 800-22 statistical tests. The random bits of 120 sequences, each with a size of 1 Mbit, were tested, and the worst *P*-value and proportion are presented. At significance level *α* = 0.01, the success proportion should be in the range of 0.99 ± 0.027, and the composite *P*-value should be larger than 0.0001 to ensure uniformity

The chaotic outputs at *I* = 25.6 mA are verified using the *G*-*P* algorithm and the calculated results are presented in Fig. 6d–f. The quantity of ln(*C*_*D*_(*ε*)) converges to a linear scale versus ln(*ε*) with increasing *D* in Fig. 6d, and the correlation dimension *ν* is converged to about 3.92. Furthermore, *K*_2_-entropy is estimated as *K*_2_ ≈ 2.9 ns^−1^ as shown in Fig. 6f, which verifies the chaotic characteristics of the laser output data. The correlation dimension *ν* of 3.92 and *K*_2_-entropy *K*_2_ ≈ 2.9 ns^−1^ are agreement very well with *ν* = 3.91 in Fig. S2d, accounting the 2 GHz bandwidth of ADC, and *K*_2_ ≈ 3.2 ns^−1^ in Fig. 3b obtained from the chaotic output series of the rate equations at Δ*f* = 10.6 GHz. Finally, random bits were generated from the temporal waveforms by retaining 2 of the least significant bits (LSBs) of each sample for the microlaser. The generated random bit sequences are verified by the National Institutes of Standards and Technology Special Publication 800-22 statistical test suite. A total of 120 sequences, each with a size of 1 Mbit, were collected and tested. As shown in Fig. 7, the random bits generated at a bit rate of 10 Gbit s^−1^ (5 GS s^−^^1^ × 2 bits) pass the test. However, random bits generated at higher bit rate cannot pass the test, such as retaining 3 of the LSBs of each sample. In addition, the size of random bit data tested is partly limited by the storage capacity of the oscilloscope. We expect to realize higher bandwidth of random bits using an oscilloscope with a larger bandwidth.

## Discussion

In summary, internal mode interaction has been proposed and demonstrated for realizing chaotic and period oscillation states without external perturbation in a dual-mode lasing microcavity laser. A rate equation model based on field equations is set up to account the internal mode interaction including the oscillations of mode beating intensity and carrier density. Dual-mode lasing of the fundamental and the first order transverse modes guarantees strong carrier oscillation inside the laser cavity due to mode beating, which enhances the mode interaction. Chaotic dual-mode lasing laser is predicted using the rate equation without external perturbation. The chaotic semiconductor microlaser and physical random number generation are demonstrated experimentally, with the results in good agreement with the theoretical prediction. The procedure in revealing the underlying mechanism of the internal interaction between two transverse modes gives a new understanding of the nonlinear dynamical process in the semiconductor microlasers. Enhancing mode *Q* factors and tuning mode intervals by using circular sides and a ring electrode pave the way for mode engineering in polygonal microcavities. The mode engineering to enhance the nonlinear dynamics in semiconductor microlasers provides stable and robust optical sources for random number generations.

## Materials and methods

### Mode characteristics simulation

The mode characteristics of the deformed hexagonal micro resonator were simulated by using a two-dimensional finite element method (FEM) (COMSOL Multiphysics 5.0) with a cell step of 25 nm. The CSHM has a constant refractive index of 3.2 corresponding to an AlGaInAs/InP laser wafer, and laterally confined by divinylsiloxane-bisbenzocyclobutene (DVS-BCB) with a refractive index of 1.54, which is coated for the planarization of the fabricated microlasers. A perfectly matched layer (PML) is used to terminate the calculation region, and the distances between the PML and the resonator edge are larger than 2 μm to ensure the accuracy of the numerical calculation.

### Experimental data acquisition

The CSHM lasers were fabricated using an AlGaInAs/InP compressively-strained multiple quantum wells laser wafer by contacted photolithography and inductively coupled plasma etching technique^38,47^. The lasers were cleaved over the output waveguide and bonded with the p-side up on an AlN submount. The optical output from the CSHM laser is collected by a single mode fiber and then analyzed by an optical spectrum analyzer (OSA, YOKOGAWA, AQ6370C) with a resolution of 0.02 nm. Alternatively, the collected beam is amplified by an EDFA and then filtered out the aimed longitudinal group (*H*_A_ or *H*_B_ depending on the interest) by a band pass filter (BPF) to reduce the influence of the other longitudinal modes. The electric signal converted from the filtered light beam by a photodetector (PD, 40 GHz bandwidth) are divided into two lines. One of the lines is measured by an electric spectrum analyzer (ESA, ROHDE&SCHWARZ, FSU, 26.5 GHz bandwidth) and the other line is measured by an oscilloscope (OSC, ROHDE&SCHWARZ, RTO1024, 2 GHz bandwidth, 10 GSa s^−1^) to measure the temporal waveform for random number generation.

### Correlation dimension and the entropy

The outputs are evaluated using the G–P algorithm to estimate the chaotic behavior^43^. To make the G–P algorithm efficient for finite time series and reduce the noise influence on the experimental data, we applied a modified version of G–P algorithm^48^. For time series data *F*(*m*) with *N* points *m* = 1 to *N*, as one-dimensional chaotic trajectory, we can reassemble the data into *D*-dimensional trajectory as *N*-*D* points of {*F*(1), *F*(2), …*F*(D)), (*F*(2), *F*(3), …*F*(*D* + 1)), …, ((*F*(*N*-*D*), *F*(*N*-*D* + 1), …*F*(N)}, with the embedding dimension of *D*. A correlation integral *C*_*D*_(*ε*) is defined as the average number of points inside a ball of radius *ε* centered at one point, where *ε* is the distance between two points in the *D-*dimensional space. *C*_*D*_(ε) will grow with the increasing of the radius as a power law of $C_D\left( \varepsilon \right) \propto \varepsilon ^\nu$. The correlation dimension *ν* and the *K*_2_-entropy are then estimated from the correlation integral curve by

2 $$\nu = {\rm{d}}\ln \left( {{{{\mathrm{C}}}}_D\left( \varepsilon \right)} \right)/{\rm{d}}\ln \left( \varepsilon \right)$$

and

3 $$K_2 = \ln \left[ {C_D\left( \varepsilon \right)/C_{D + 1}\left( \varepsilon \right)} \right]/\tau$$

as *D* and *ε* approach infinity and zero, respectively, where *τ* is the sampling rate of the time series. The number of temporal points used for the G–P algorithm is 4000 for both of the simulation and experimental data. For re-embedding procedure^48^, we use a window size of 12 and three principal components. Generally, infinite is out of range numerically, but these functions show good convergence when the embedding dimension *D* is larger than 2*υ* + 1^49^. So, we look for convergence in these functions as we increase the vector size *D*.

## Supplementary information

Supplementary_materials

## References

[1] Gisin N (2002). Quantum cryptography. Rev. Mod. Phys..

[2] Herrero-Collantes M, Garcia-Escartin JC (2017). Quantum random number generators. Rev. Mod. Phys..

[3] Liu Y (2018). High-speed device-independent quantum random number generation without a detection loophole. Phys. Rev. Lett..

[4] Asmussen, S. & Glynn, P. W. *Stochastic Simulation: Algorithms and Analysis* (Springer, 2007).

[5] Maddocks RS (1972). A compact and accurate generator for truly random binary digits. J. Phys. E: Sci. Instrum..

[6] Holman WT, Connelly JA, Dowlatabadi AB (1997). An integrated analog/digital random noise source. IEEE Trans. Circuits Syst. I: Fundamental Theory Appl..

[7] Tokunaga C, Blaauw D, Mudge T (2008). True random number generator with a metastability-based quality control. IEEE J. Solid-State Circuits.

[8] Kanter I (2010). An optical ultrafast random bit generator. Nat. Photonics.

[9] Uchida A (2008). Fast physical random bit generation with chaotic semiconductor lasers. Nat. Photonics.

[10] Mukai T, Otsuka K (1985). New route to optical chaos: Successive-subharmonic-oscillation cascade in a semiconductor laser coupled to an external cavity. Phys. Rev. Lett..

[11] Zhang LM (2017). 640-Gbit/s fast physical random number generation using a broadband chaotic semiconductor laser. Sci. Rep..

[12] Mørk J, Mark J, Tromborg B (1990). Route to chaos and competition between relaxation oscillations for a semiconductor laser with optical feedback. Phys. Rev. Lett..

[13] Li NQ (2014). Two approaches for ultrafast random bit generation based on the chaotic dynamics of a semiconductor laser. Opt. Express.

[14] Reidler I (2009). Ultrahigh-speed random number generation based on a chaotic semiconductor laser. Phys. Rev. Lett..

[15] Butler T (2016). Optical ultrafast random number generation at 1 Tb/s using a turbulent semiconductor ring cavity laser. Opt. Lett..

[16] Schikora S, Wünsche HJ, Henneberger F (2008). All-optical noninvasive chaos control of a semiconductor laser. Phys. Rev. E.

[17] Wu JG (2013). Direct generation of broadband chaos by a monolithic integrated semiconductor laser chip. Opt. Express.

[18] Argyris A (2010). Implementation of 140 Gb/s true random bit generator based on a chaotic photonic integrated circuit. Opt. Express.

[19] Argyris A (2008). Photonic integrated device for chaos applications in communications. Phys. Rev. Lett..

[20] Simpson TB (1995). Period-doubling cascades and chaos in a semiconductor laser with optical injection. Phys. Rev. A.

[21] Sciamanna M, Shore KA (2015). Physics and applications of laser diode chaos. Nat. Photonics.

[22] Argyris A (2005). Chaos-based communications at high bit rates using commercial fibre-optic links. Nature.

[23] Larger L, Dudley JM (2010). Optoelectronic chaos. Nature.

[24] Ke JX (2018). Chaotic optical communications over 100 km fiber transmission at 30-Gb/s bit rate. Opt. Lett..

[25] Uchida, A. *Optical Communication with Chaotic Lasers: Applications of Nonlinear Dynamics and Synchronization* (Wiley‐VCH Verlag GmbH & Co. KGaA, 2012).

[26] Li XZ, Chan SC (2012). Random bit generation using an optically injected semiconductor laser in chaos with oversampling. Opt. Lett..

[27] Zou LX (2015). Integrated semiconductor twin-microdisk laser under mutually optical injection. Appl. Phys. Lett..

[28] Rontani D (2007). Loss of time-delay signature in the chaotic output of a semiconductor laser with optical feedback. Opt. Lett..

[29] Albert F (2011). Observing chaos for quantum-dot microlasers with external feedback. Nat. Commun..

[30] Virte M (2013). Deterministic polarization chaos from a laser diode. Nat. Photonics.

[31] Miguel MS, Feng Q, Moloney JV (1995). Light-polarization dynamics in surface-emitting semiconductor lasers. Phys. Rev. A.

[32] Bittner S (2018). Suppressing spatiotemporal lasing instabilities with wave-chaotic microcavities. Science.

[33] Kim K (2021). Massively parallel ultrafast random bit generation with a chip-scale laser. Science.

[34] Cao H, Wiersig J (2015). Dielectric microcavities: Model systems for wave chaos and non-Hermitian physics. Rev. Mod. Phys..

[35] Wang S (2021). Direct observation of chaotic resonances in optical microcavities. Light.: Sci. Appl..

[36] Qian YJ (2021). Regulated photon transport in chaotic microcavities by tailoring phase space. Phys. Rev. Lett..

[37] Long H (2015). Dual-transverse-mode microsquare lasers with tunable wavelength interval. Opt. Lett..

[38] Xiao ZX (2017). Single-mode unidirectional-emission circular-side hexagonal resonator microlasers. Opt. Lett..

[39] Kawaguchi H (1984). Optical bistability and chaos in a semiconductor laser with a saturable absorber. Appl. Phys. Lett..

[40] Bracikowski C, Roy R (1991). Chaos in a multimode solid-state laser system. Chaos: Interdiscip. J. Nonlinear Sci..

[41] Erzgräber H, Wieczorek S, Krauskopf B (2008). Dynamics of two laterally coupled semiconductor lasers: Strong- and weak-coupling theory. Phys. Rev. E.

[42] Vaughan M (2019). Stability boundaries in laterally-coupled pairs of semiconductor lasers. Photonics.

[43] Grassberger P, Procaccia I (1983). Estimation of the kolmogorov entropy from a chaotic signal. Phys. Rev. A.

[44] Grassberger P, Procaccia I (1983). Characterization of strange attractors. Phys. Rev. Lett..

[45] Hao YZ (2021). Comparison of single- and dual-mode lasing states of a hybrid-cavity laser under optical feedback. Opt. Lett..

[46] Someya H (2009). Synchronization of bandwidth-enhanced chaos in semiconductor lasers with optical feedback and injection. Opt. Express.

[47] Xiao, J. L. et al. Random bit generation in dual transverse mode microlaser without optical injection or feedback. In *Proceedings of 2018 IEEE International Semiconductor Laser Conference* 171–172 (IEEE, 2018).

[48] Fraedrich K, Wang RS (1993). Estimating the correlation dimension of an attractor from noisy and small datasets based on re-embedding. Physica D: Nonlinear Phenom..

[49] Holzfuss, J. & Mayer-Kress, G. An approach to error-estimation in the application of dimension algorithms. In *Proceedings of Quantification of Complex Behavior Proceeding of an International Workshop* 114–122 (Springer, 1986).

