# Supplementary material for: Chaotic microlasers caused by internal mode interaction for random number generation
Source: Light Sci Appl. 2022 Jun 20;11:187. doi: 10.1038/s41377-022-00890-w (PMC9209477; doi:10.1038/s41377-022-00890-w)
Supplement: Supplementary file 1 — Supplementary_materials [file 41377_2022_890_MOESM1_ESM.docx]

Supplementary Information for

Chaotic Microlasers Caused by Internal Mode Interaction for Random Number Generation

Chun-Guang Ma^1,2,#^, Jin-Long Xiao^1,2,#^, Zhi-Xiong Xiao^1,2^, Yue-De Yang^1,2^, and Yong-Zhen Huang^1,2^,*

^1^ State Key Laboratory of Integrated Optoelectronics, Institute of Semiconductors, Chinese Academy of Sciences, Beijing 100083, China

^2^ Center of Material Science and Optoelectronic Engineering, University of Chinese Academy of Sciences, Beijing 100049, China

#These authors contributed equally to this work

[*Corresponding author: yzhuang@semi.ac.cn](mailto:*yzhuang@semi.ac.cn)

1. Rate equation model

We rewrite the rate equation Eq. (1) in the form of $\left|E_{m}(t)\right|$and $\Phi_{m}(t)$with mode electric field$E_{m}(t)=\left|E_{m}(t)\right|\exp(j\Phi_{m}(t))$, where is the varying phase of mode *m*, $\Omega$is a reference frequency.is the phase difference between mode *m* and *m*’, and the mode frequency interval $\Delta f=\frac{1}{2\pi}(\omega_{1}-\omega_{0})$.

(S1)

The fundamental and the first order transverse modes are considered as orthogonal modes in the CSHM with a constant refractive index distribution. However, the refractive index varies with the oscillation of the carrier density, which may lead to an interaction between the modes. To characterize the different superpositions of the H_0_ and H_1_ modes in different areas, the cross coefficient *κ* for the two modes are calculated and plotted in Fig. 1(d) according to

$\kappa(x,y)\text{=}\frac{H_{0}(x,y)H_{1}^{*}(x,y)}{\sqrt{\int\left|H_{0}(x,y)\right|^{2}dxdy\cdot\int\left|H_{1}(x,y)\right|^{2}dxdy}}\text{ }$ (S2)

Clear distributions of the in-phase and anti-phase sections (*A* and *B*) can be observed in Fig. 1(d), which are similar with the two modes in the two-coupled cavity in Ref.[[1](#_ENREF_1)]. The spatial mode integral for the two modes over *A* and *B* is calculated by:

$K_{mm'}^{A,B}\text{=}\frac{\int_{A,B} H_{m}(x,y)H_{m'}^{*}(x,y)dxdy}{\sqrt{\int\left|H_{m}(x,y)\right|^{2}dxdy\cdot\int\left|H_{m'}(x,y)\right|^{2}dxdy}}\text{ }$ (S3)

The *A* (*B*) section is composed by the areas with *κ* > 0 (*κ* < 0), which oscillates at a period *T* inversely proportion to the mode frequency interval. *H_m_*(*x*, *y*) [*H_m_*’(*x*, *y*)] is the mode magnetic-field distributions of transverse mode *m* (*m*’) derived from the simulated results in the inset of Fig. 1(c). Considering the spatial dependance of the carrier density on *A* and *B*, the gain coefficients for the modes in the *A* and *B* sections can be expressed as:

$g^{A,B}=\frac{g_{0}}{1+\zeta(\left|E_{0}\right|^{2}+\left|E_{1}\right|^{2})}\ln\left(\frac{N^{A,B}+N_{s}}{N_{tr}+N_{s}}\right)$ (S4)

The self- and cross- gain suppression coefficients are taken the same value of *ζ*. *N^A,B^* represents the carrier density in the *A* and *B* sections. The spatial mode integral and the other parameters used in the rate equation models are listed in Table S1. As the in-phase and anti-phase sections alternate with each other at the period *T*, we neglect the effect of carrier diffusion in the rate equation. The photon density of the output in the simulation is defined as . The extremum-bifurcation diagram in Fig. 2(b) is calculated by searching for the local maxima and minima of *S* from the output with a length of 100 ns at different frequency intervals of the two modes. Different local maxima (minima) are distinguished only if their values have a relative difference larger than 10^-3^.

Table S1. Parameters used in rate equations for the CSHM

| Parameters | Definition | Value |
| --- | --- | --- |
| *N_tr_* | Transparency density | 1.2×10^18^ cm^-3^ |
| *N_s_* | Logarithmic gain parameter | 0.092 *N_tr_* |
| *n_g_* | Group refractive index | 3.5 |
| *η* | Current injection efficiency | 0.7 |
| *α* | Linewidth enhancement factor | 3 |
| *β* | Spontaneous emission factor | 10^-2^ |
| *Γ* | Confinement factor | 0.2 |
| *A* | Defect recombination coefficient | 10^8^ s^-1^ |
| *B* | Bimolecular recombination coefficient | 10^-10^ cm^3^s^-1^ |
| *C* | Auger recombination coefficient | 10^-28^ cm^6^s^-1^ |
| *Q*_0_ (*Q*_1_) | Passive mode quality factor of H_0_ (H_1_) | 8600(5400) |
| *α_i_* | Internal absorption loss | 20 cm^-1^ |
| *g_0_* | Material gain parameter | 1500 |
| ζ | Gain suppression factor | 1.5×10^-17^ cm^3^ |
| *K^A^_mm’_* (*K^B^_mm’_*) (*m* ≠ *m*’) | Cross-spatial mode integral | 0.3 (-0.3) |
| *K*_00_ (*K*_11_) | Self-spatial mode integral | 1 |
| *I* | Injection current | 15 mA |


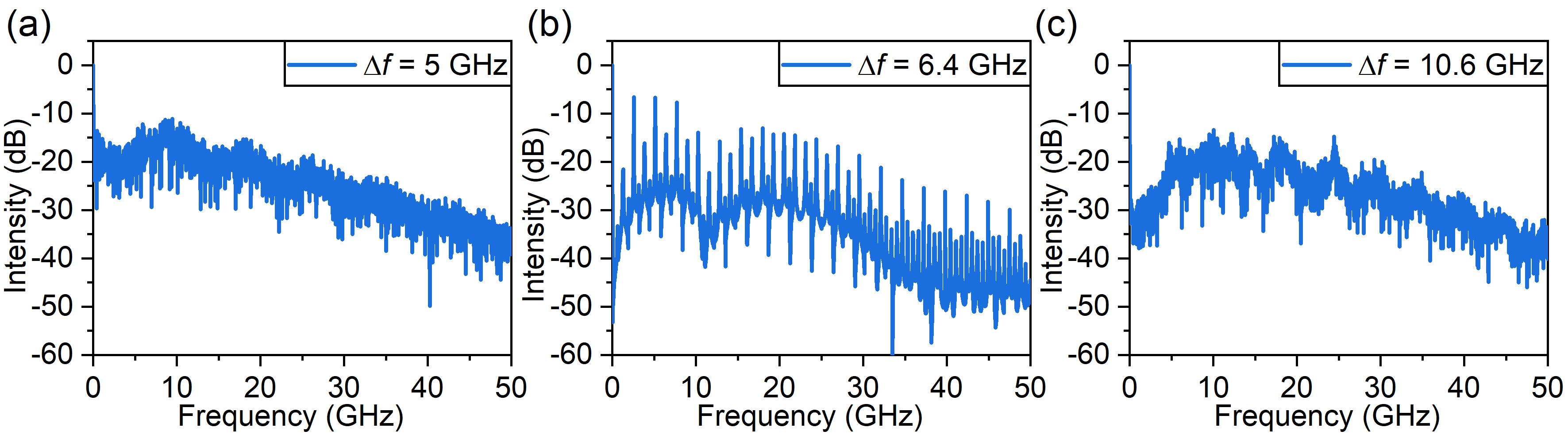


**Fig. S1. Numerical results of RF spectra at the mode frequency intervals of** (a) 5 GHz (b) 6.4 GHz (c) 10.6 GHz.

The numerical results of output RF spectra are presented in Fig. S1 at the mode frequency intervals of 5, 6.4, and 10.6 GHz, respectively. As shown in Fig. S1 (a) at Δ*f* = 5 GHz, the RF spectra have a peak around 10 GHz near the resonance frequency *f_r_*, which is even larger than that in Fig. S1(c) at Δ*f* = 10 GHz. The results indicate that Δ*f = f_r_*/2 can still have strong resonance peak around *f_r_*, which can explain the chaotic region near Δ*f* = 5 GHz in Fig. 2(b) in addition to the chaotic region around the resonance frequency. In addition, multiple peaks are observed in the RF spectra in Fig. S1(b) at Δ*f* = 6.4 GHz.

2. Correlation dimension and the entropy

Comparing the simulated results in Fig. 2(e), the slopes of the integral curves fluctuate at small radius in Fig. 6(e) for experimental results, because the “points” in the ball become less with the reduction of the radius. The condition that the number of “points” should be infinite is not satisfied anymore, leading to discrete changing of the integral curve [[2](#_ENREF_2)]. Besides, the radius in fact represents the intensity scale, because the *D*-dimensional-point-space is constructed by reassembling the one-dimensional time series. Thus, for experimental data, the noise becomes dominating at small radius, resulting in a smaller range of convergence than the simulation data in the correlation integral curve. As shown in Fig. S2(a), the optical output of the laser is first converted to electric signal by a photodetector and then measured using an oscilloscope with the analog to digital converter (ADC) of a 2 GHz bandwidth in the experiment. We now consider the influence of the bandwidth of the ADC on the numerical results of rate equations. The simulated analog electric signal was converted to 8-bit digital signal by an 8-bit ADC with 2 GHz bandwidth. As shown in Fig. S2(b), the waveform of the original signal is almost the same as that after the ADC, which is obtained by dividing the amplitudes of the original waveform into 256 discrete levels to play the function of ADC. The obtained digital discrete waveform is utilized for calculating the correlation integral and Kolmogrovo-entropy. The correlation integral of the filtered simulation signal is shown in Fig. S2(c) and the slope of the correlation integral is shown in Fig. S2(d), which is convergent to a correlation dimension *ν* ≈ 3.91. The results show good agreement with the experimental ones compared to Figs. 6(d) and 6(e), which indicates that the bandwidth of the ADC has a big influence on the reconstruction of the signal and thus the computation of the correlation dimension.


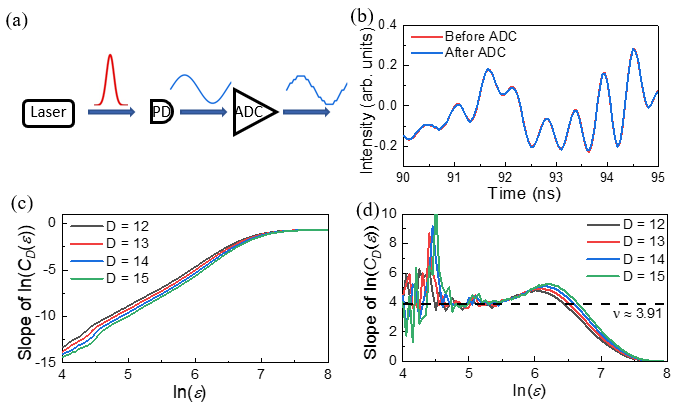


**Fig. S2. The process of the timeseries acquisition and the effect on the estimation of the Kolmogrovo-entropy.** (a)Schematic diagram of the signal measurement process. (b) Simulated output analog (red curve) and 8-bit digital timeseries to show the influence of the analog to digital process. (c) Correlation integral and (d) the slope of the correlation integral of the numerical timeseries of the rate equations accounting the 2 GHz bandwidth of the ADC.

References

1. Erzgraber, H., Wieczorek, S. & Krauskopf, B. Dynamics of two laterally coupled semiconductor lasers: Strong- and weak-coupling theory. *Physical Review E* **78**, 066201 (2008).

2. Grassberger, P. & Procaccia, I. Estimation of the kolmogorov-entropy from a chaotic signal. *Physical Review A* **28**, 2591-2593 (1983).
